# Multiplex single‐cell profiling of putative cancer stem cell markers ALDH1, SOX9, SOX2, CD44, CD133 and CD15 in endometrial cancer

**DOI:** 10.1002/1878-0261.13815

**Published:** 2025-01-31

**Authors:** Hilde E. Lien, Marta E. Hjelmeland, Hege F. Berg, Rose M. Gold, Kathrine Woie, Lars A. Akslen, Ingfrid S. Haldorsen, Camilla Krakstad

**Affiliations:** ^1^ Department of Clinical Science, Centre for Cancer Biomarkers CCBIO University of Bergen Norway; ^2^ Department of Gynecology and Obstetrics Haukeland University Hospital Bergen Norway; ^3^ Department of Clinical Medicine, Centre for Cancer Biomarkers CCBIO University of Bergen Norway; ^4^ Department of Pathology Haukeland University Hospital Bergen Norway; ^5^ Department of Radiology, Mohn Medical Imaging and Visualization Centre Haukeland University Hospital Bergen Norway; ^6^ Section for Radiology, Department of Clinical Medicine University of Bergen Norway

**Keywords:** cancer stem cells, endometrial cancer, imaging mass cytometry, patient‐derived organoids, single cell, tumor heterogeneity

## Abstract

The presence of cancer stem cells is linked to aggressive disease and higher risk of recurrence, and multiple markers have been proposed to detect cancer stem cells. However, a detailed evaluation of the expression patterns and the prognostic value of markers relevant for endometrial cancer is lacking. As organoid models are suggested to be enriched in cancer stem cells, such models may prove valuable to define tissue‐specific cancer stem cells. To address this, imaging mass cytometry and multiplex single‐cell analyses were performed on an endometrial cancer patient series including both tumor biopsies and corresponding patient‐derived organoids. An antibody panel focused on cancer stem cell markers was used to identify cancer stem cell phenotypes. Over 70% of epithelial cells in the tumor biopsies expressed at least one putative cancer stem cell marker. We identified distinct cancer cell phenotypes with heterogeneous expression within individual patients and between patient samples. Few differences in the distribution of cancer cell phenotypes were observed between tumor biopsies and corresponding organoids. Cells expressing aldehyde dehydrogenase 1 (ALDH1) were more prevalent in high‐grade tumors, while expression of CD44 was more prevalent in grade 1 tumors. Spatial analysis revealed significantly less interaction between ALDH1‐ and CD44‐expressing cells. Gene expression data was used to further investigate selected markers. *CD44* gene expression was associated with a favorable prognosis and was further validated using immunohistochemistry. High expression of CD44 was significantly associated with better survival. The general high expression of proposed stem cell markers may indicate alternative roles for these in endometrial cancer.

## Graphical abstract

Cancer stem cells are associated with aggressive disease, but a deep characterization of such markers is lacking in endometrial cancer. This study uses imaging mass cytometry to explore putative cancer stem cell markers in endometrial tumors and corresponding organoid models. Analyses showed a high number of cancer cells expressing stemness markers. CD44 expression associated with patient prognosis.

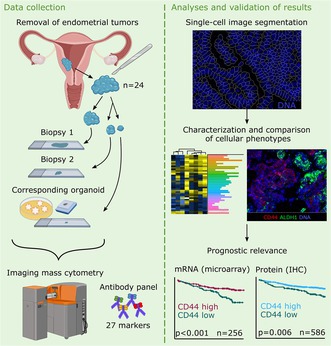

**Abbreviations**
CD: cluster of differentiation
CNH: copy number high
CNL: copy number low
ER: estrogen receptor
FFPE: formalin‐fixed paraffin embedded
FIGO: international federation of gynecology and obstetrics
FUT4: fucosyltransferase 4 (CD15)
IHC: immunohistochemistry
IMC: imaging mass cytometry
MEM: marker enrichment modeling
MMR: mismatch repair
NEC: non‐endometrioid endometrial cancer
POLE: polymerase ε catalytic subunit ultramutated
PR: progesterone receptor
PROM1: prominin 1 (CD133)
SI: staining index
SOX2: sex determining region Y‐like box transcription factor 2
SOX9: sex determining region Y‐like box transcription factor 9
TBS: tris‐buffered saline
TMA: tissue microarray
UMAP: Uniform Manifold Approximation and Projection
αSMA: α smooth muscle actin

## 1 Introduction

Endometrial cancer is the sixth most common cancer type in women worldwide [1] with two main histologic subtypes, endometrioid (80%) and non‐endometrioid (20%) tumors. Non‐endometrioid tumors are more aggressive and associated with worse prognosis. Molecular profiling of endometrial cancers has identified four subgroups of tumors, POLE ultramutated (POLE), mismatch repair (MMR) deficient, copy number low (CNL) and copy number high (CNH) [2]. When diagnosed at an early stage, most patients are disease‐free after hysterectomy, however, 40–60% of patients with advanced or metastatic disease experience treatment resistance and disease progression [3].

A subpopulation of cancer cells with stem‐like characteristics has been linked to aggressive disease, recurrence and treatment resistance [4]. These cancer stem cells are self‐renewing, can divide asymmetrically and give rise to differentiated cancer cells. These self‐renewing properties also support an enrichment of cancer stem cells in patient‐derived organoid models that can be cultured for a prolonged time [5]. These models recapitulate the genetic, transcriptomic and phenotypic characteristics of the original tissue and have successfully been established from endometrial cancer tissue [6, 7, 8]. Cancer stem cells have been referred to as tumor‐initiating cells and have been proposed as possible treatment targets [9, 10].

Numerous cancer stem cell markers have been reported for multiple types of cancer [11] but few reports have investigated expression patterns of established cancer stem cell markers in endometrial cancer. A comprehensive characterization of proposed cancer stem cell markers can provide valuable information on their relevance in endometrial cancer as well as their potential as prognostic markers. We therefore aimed to define the spatial localization and prognostic relevance of putative endometrial cancer stem cell markers. Multiplex single‐cell protein detection by imaging mass cytometry (IMC) was performed in a unique collection of paired endometrial cancer tumors and organoids to investigate the presence of several previously proposed cancer stem cell markers. An antibody panel including epithelial, stromal, immune and cancer stem cell markers was designed to enable both cell type identification (epithelial and non‐epithelial) and a more detailed examination of the expression and distribution of cancer stem cell markers. The panel included previously reported endometrial cancer stem cell markers [12, 13, 14] (CD44, ALDH1 and CD133), normal endometrial stem cell markers [15] (SOX9 and CD15) and universal stem cell marker [16]. All included markers have also been reported as cancer stem cell markers in other cancer types [9, 10, 11, 17]. In general, the cancer stem cell markers are all linked to stem cell signaling, tumorigenesis and therapy resistance [18, 19, 20, 21, 22, 23]. Pinpointing endometrial cancer stem cell markers would add valuable insight regarding stem cells that may prove relevant for risk stratification of cancer patients and guide further research on targeted treatment.

## 2 Materials and methods

### 2.1 General biobanking and approvals

Tumor tissue was prospectively obtained from patients diagnosed with endometrial cancer and treated at Haukeland University Hospital, Bergen, Norway from 2001 to 2015. Clinical data and histopathological characteristics were retrieved from patient records and routine pathology reports. All patients included in the study gave written and informed consent. Samples were stored at the Bergen Biobank for gynecological cancer (REK 2014/1907) and patient information was stored in the Bergen gynecological cancer health registry (approved by the Norwegian Data Inspectorate 2016/7421 and Regional ethical committee, REK 7226). The present study has been approved by the regional ethical committee (REK 2018/594). The study is conformed to the standards of the declaration of Helsinki.

### 2.2 Patient cohort for imaging mass cytometry

For IMC, a subset of 24 patients from the prospectively collected biobank cohort was included. The patients were selected based on the successful establishment of an organoid model from the corresponding patient tissue. When available, two formalin‐fixed paraffin‐embedded (FFPE) tissue sections from the primary tumor were collected from each patient. One section represented the pathology biopsy (Biopsy 1), used for diagnosis and one section represented the biobank biopsy (Biopsy 2). In addition, one FFPE section of a corresponding patient‐derived organoid sample was collected from each patient. For nine patients, the organoids were cultured for a longer time period (up to 6 months) and a second FFPE section of the late organoid culture was collected. In total, 77 sections were stained for IMC where five patients were represented by four FFPE sections each (biopsy 1, biopsy 2, organoid early and organoid late), 15 patients were represented by three FFPE sections (biopsy 1, biopsy 2 and organoid early), three patients were represented by three FFPE sections (biopsy 1, organoid early and organoid late) and one patient was represented by three FFPE sections (biopsy 1, biopsy 2 and organoid late). Clinical characteristics of the IMC patient cohort are found in Table S1.

### 2.3 Patient‐derived organoids

Patient‐derived organoids were developed as previously described [6]. Briefly, fresh tumor tissue was incubated in custom‐made media for 20 min at 37 °C and 5% (v/v) CO_2_ prior to mechanistic dissociation and enzymatic digestion of the cancer cells. To remove undigested tissue, the cancer cells were filtered through a 100 μm Nylon cell strainer (431 752; Corning, Corning, NY, USA) and subsequently washed in red blood cell lysis buffer. The cancer cells were embedded in growth factor reduced Matrigel (354 230; Corning) and seeded as droplets into 48‐well plates before being covered in an optimized expansion media.

### 2.4 Patient cohort for immunohistochemistry

For immunohistochemical (IHC) protein expression evaluation, a subset of the biobank cohort was used. Tissue microarrays (TMAs) of 586 patients were stained for CD44.

### 2.5 Antibody conjugation and validation for imaging mass cytometry

Antibody conjugation was performed using the Maxpar Antibody labeling kits (Standard BioTools, South San Francisco, CA, USA), following the manufacturer's protocol. The concentration of conjugated antibodies was measured using NanoDrop (RRID:SCR_016517; Thermo Fisher Scientific, Waltham, MA, USA) then diluted to 0.5 mg/mL in Antibody stabilizer (131 050; CANDOR Bioscience GmbH, Wangen, Germany) and stored at 4 °C. In‐house conjugated antibodies were tested after conjugation with IHC on endometrial cancer tissue to check the specificity of the staining. Pre‐conjugated and in‐house conjugated antibodies were tested with IMC to define optimal staining concentration on endometrial cancer tissue. The list of antibodies for IMC are found in Table S2.

### 2.6 Imaging mass cytometry staining

FFPE sections were incubated at 60 °C for 2 h before being deparaffinized in xylene, rehydrated in a series of ethanol (100%, 95%, 80%, 70% and 50%) and washed in MilliQ water. For antigen retrieval, sections were boiled for 30 min in Tris buffer (pH 9, Dako), then washed in MilliQ water and Tris‐buffered saline (TBS, 2 × 10 min) before blocking using Superblock (37 581; Thermo Scientific) for 30 min. To stain the 77 tissue sections, the sections were divided into four batches (20–22 sections per batch) to avoid procedural delays during the staining process. A panel of metal‐tagged antibodies (Table S2) was mixed and divided into four batches. The antibody cocktail batches were frozen at −80 °C to avoid aberrant staining patterns between batches. BSA 1% Triton 0.1% in TBS were added to the thawed antibody cocktail prior to staining. Sections were stained over night at 4 °C and then washed in 0.1% triton 100‐x TBS and TBS (2 × 8 min). For visualization of DNA, sections were stained with Intercalator‐Ir (1:500; Standard BioTools, 201192A). Sections were washed in MilliQ water and airdried for 20 min at room temperature before ablation using the Hyperion Imaging System (Standard BioTools, RRID:SCR_023195) at a frequency of 200 hz. Two regions of interest were defined on hematoxylin and eosin‐stained sections and used to guide the selection of ablation area in each slide for IMC. No differences were found in median signal intensity of all markers per section between batches after ablation (Kruskal‐Wallis, *P* = 0.12).

### 2.7 Image and data processing

MCD viewer (v.1.0.560.6, Standard BioTools, RRID:SCR_023007) was used to visualize and export images of raw IMC files. Single cells were extracted using the Steinbock framework (v.0.16.0) [24]. Selected markers of cell membrane, cytoplasm and nuclei were used by the algorithm to create masks of each single cell. Non‐cell objects identified as cells, mainly due to hot pixels in the DNA channels, were manually removed from the dataset prior to analysis. Images and corresponding cell masks were visualized in histocat (v.1.76). Single‐cell data expression values were arcsinh transformed with a cofactor of 1 in R/rstudio (R v.4.3.0, rstudio v.2022.07.0, RRID:SCR_001905) and downstream analysis was performed in R/rstudio.

### 2.8 Defining tumor biopsy cell type composition

Tumor biopsy composition was determined by an initial phenograph clustering (rphenograph package v.0.99.1, RRID: SCR_022603) of all tumor biopsy cells based on the following markers: pan‐cytokeratin, vimentin, E‐cadherin, αSMA, ER, PR, β‐catenin, VEGF, pS6, pERK1/2, collagen type I, p53, ki‐67, podoplanin, cleaved caspase 3, CD8, CD3, CD4, CD20, CD68 and CD31. The target of each marker is found in Table S2. The clusters were annotated into epithelial and non‐epithelial (stromal and immune cells) based on marker enrichment modeling (MEM v.2.0.0, RRID: SCR_022495) and hierarchical clustering of the phenograph clusters. T cell clusters were identified by positive expression of CD4 or CD8 and the pan T cell marker CD3. Epithelial cells were identified by the expression of E‐cadherin, pan‐cytokeratin and lack of αSMA expression. Here, epithelial cells denote epithelial cancer cells in endometrioid, serous and clear cell tumors and all cancer cells from carcinosarcomas.

### 2.9 Defining tumor biopsy and organoid epithelial phenotypes

Distinct epithelial phenotypes were identified by phenograph clustering of tumor biopsy epithelial cells and organoid cells together. Organoids contained only epithelial cells and epithelial cells identified in primary tumor biopsies were analyzed together with organoid cells. The initial clustering was based on marker expression of pan‐cytokeratin, vimentin, E‐cadherin, αSMA, ER, PR, β‐catenin, VEGF, pS6, pERK1/2, collagen type I, p53, Ki‐67, podoplanin and cleaved caspase 3. The phenograph clusters were merged into 29 phenotypes based on similar expression patterns identified by MEM and hierarchical clustering.

### 2.10 Defining cancer stem cell phenotypes

To characterize cells expressing cancer stem cell‐associated markers, epithelial cells from primary tumors and organoids were clustered using phenograph separately to avoid under‐clustering of rare cancer stem cell phenotypes. The cells were clustered based on the markers ALDH1, CD44, SOX2, SOX9, CD133 and CD15. The initial phenograph clusters were annotated into 12 phenotypes in the tumor biopsies and 15 phenotypes in the organoids based on MEM and hierarchical clustering, where 11 phenotypes were shared between the biopsies and the organoids. The identified phenotypes are referred to as cancer stem cell phenotypes in this study and cells not expressing any cancer stem cell markers are referred to as low. To make sure the heterogeneity of the cancer stem cell phenotypes was not affected by the abundance of epithelial cells, we performed a correlation of the stroma/epithelial ratio against the number of cancer stem cell phenotypes present in each sample. No correlation of the stroma/epithelial ratio and cancer stem cell heterogeneity was observed (*R* = −0.1, *P* = 0.64).

### 2.11 Spatial interaction analyses

Spatial interaction analyses of cancer stem cell phenotypes, T cells (CD8/CD4) and stromal cells were performed in R/Rstudio based on the spatial analysis function in the IMC Data Analysis Workflow [25] using the R packages SpatialExperiment (v.1.10.0) and imcrtools (v.1.6.4). Spatial analysis was used to detect spatial interactions between cancer stem cell phenotypes, T cells and stromal cells, and was performed separately for tumor biopsies and organoids. This is due to the different growth conditions of organoid cells and lack of immune and stromal cells in culture.

### 2.12 Gene expression data and analyses

A previously generated microarray gene expression dataset from 256 endometrial tumors [26] was used to compare gene expression of cancer stem cell markers used in the IMC dataset. For survival analyses, gene expression data was ranked in quartiles and investigated for prognostic potential in Kaplan–Meier analyses. Best cut‐off was manually decided based on separation of outcome. In final analyses, the lowest quartile was defined as low expression and the remaining values as high expression for *CD44*, *ALDH1A1* and *FUT4* (CD15). For *PROM1* (CD133) and *SOX2*, the highest quartile was defined as high expression and the rest defined as low expression. For *SOX9*, median value was used to define high (above median) and low (less than median) expression.

For validation of results, mRNA data of endometrial tumors from the PanCancer Atlas dataset (Uterine Corpus Endometrial Carcinoma (TCGA, PanCancer Atlas), *n* = 529) was downloaded from cBioportal [27, 28] and expression of *CD44* explored. Three cases were censored due to lack of follow‐up data for progression‐free survival and five cases were censored due to lack of data for disease‐specific survival. For survival analyses, gene expression data was ranked similar to the in‐house microarray data for *CD44*, the lowest quartile was defined as low expression while the three highest quartiles were defined as high expression.

### 2.13 Tissue microarray

TMAs for IHC evaluation were constructed from hysterectomy specimens, as previously described [29]. Briefly, the area of the highest tumor grade was identified on hematoxylin and eosin‐stained full sections and three tissue cylinders of 0.6 mm in diameter were punched out. The cylinders were mounted on a new paraffin block using a custom‐made precision instrument (Beecher Instruments, Estigen, Tartu, Estonia).

### 2.14 Immunohistochemistry

IHC was performed after minor modifications of a previously described standard protocol [30, 31]. Briefly, TMA sections of the cancer tissues were deparaffinized in xylene and rehydrated in a series of ethanol solutions (100%, 96% and 80%). Antigen retrieval was performed by boiling the sections for 15 min in an antigen retrieval buffer at pH9 (Dako, S2367) before peroxidase blocking (Dako, S2023) for 8 min. The sections were incubated for 1 h at room temperature with the primary antibody mouse anti‐human CD44 (1:30, DF1485, Agilent Technologies, Santa Clara, CA, USA, M7082, RRID: AB_2076596). The sections were then incubated with anti‐mouse (K4001; Agilent Technologies) horseradish peroxidase (HRP)‐conjugated secondary antibody for 30 min. Sections were incubated with diaminobenzidine peroxidase (DAB‐chromogen; K3468, EnVision Detection System) for 5 min and counterstained with Hematoxylin (Dako, S3301) before dehydration and mounting.

### 2.15 Evaluation of immunohistochemistry staining

Protein expression of CD44 by IHC was evaluated using the semi‐quantitative staining index (SI) scoring method as previously described [31, 32]. SI was calculated by multiplying a staining intensity score (loss = 0, weak = 1, moderate = 2, strong = 3) with the percent area of positive stained tumor tissue (<10% = 1, 10–50% = 2, >50% = 3). Scoring of the tissue was performed blinded for clinical characteristics and only staining of tumor cells was evaluated. In survival analyses, SI scores for CD44 were ranked in quartiles and best cut‐off was determined based on separation of outcome from Kaplan–Meier analyses. Statistics were performed between two groups, lowest quartile (“low”; SI = 0 and 1) versus the rest (“high”; SI = 2, 3, 4, 6 and 9) expression.

### 2.16 Molecular classification

Molecular subgroup classification was defined by *POLE* sequencing and IHC of p53 and MMR proteins. Sanger sequencing was used to determine pathogenic mutations in exons 9, 11, 13 and 14 of *POLE*, a catalytic subunit of DNA polymerase‐ε. IHC was used to determine loss or intact expression of the MMR proteins: MLH1, PMS2, MSH2 and MSH6, in addition to normal or abnormal expression of p53. IHC and Sanger sequencing was performed as previously described [6]. The POLE group was defined by presence of POLE mutation, MMR deficiency was defined as loss of one or more of the MMR proteins. CNL was defined as POLE wild type, MMR proficient and normal p53 expression. Tumors with abnormal expression of p53 (loss or overexpression) were classified as CNH.

### 2.17 Statistical analysis

Statistical analyses were performed with SPSS (v.28, IBM, RRID:SCR_002865) and R/Rstudio. Categorical variables were correlated using the Fisher's exact test and continuous variables were compared using Mann–Whitney U test. The Kaplan–Meier method was used to analyze disease‐specific survival where the difference was calculated using log‐rank test (Mantel–Cox). Disease‐specific survival was calculated from the date of primary treatment to the date of death from disease. Patients who died from other causes or were lost during follow‐up time were censored. *P*‐values were two sided and considered significant if *P* < 0.05.

## 3 Results

### 3.1 Cancer stem cell markers are widely expressed in endometrial cancer samples

**Fig. 1:**
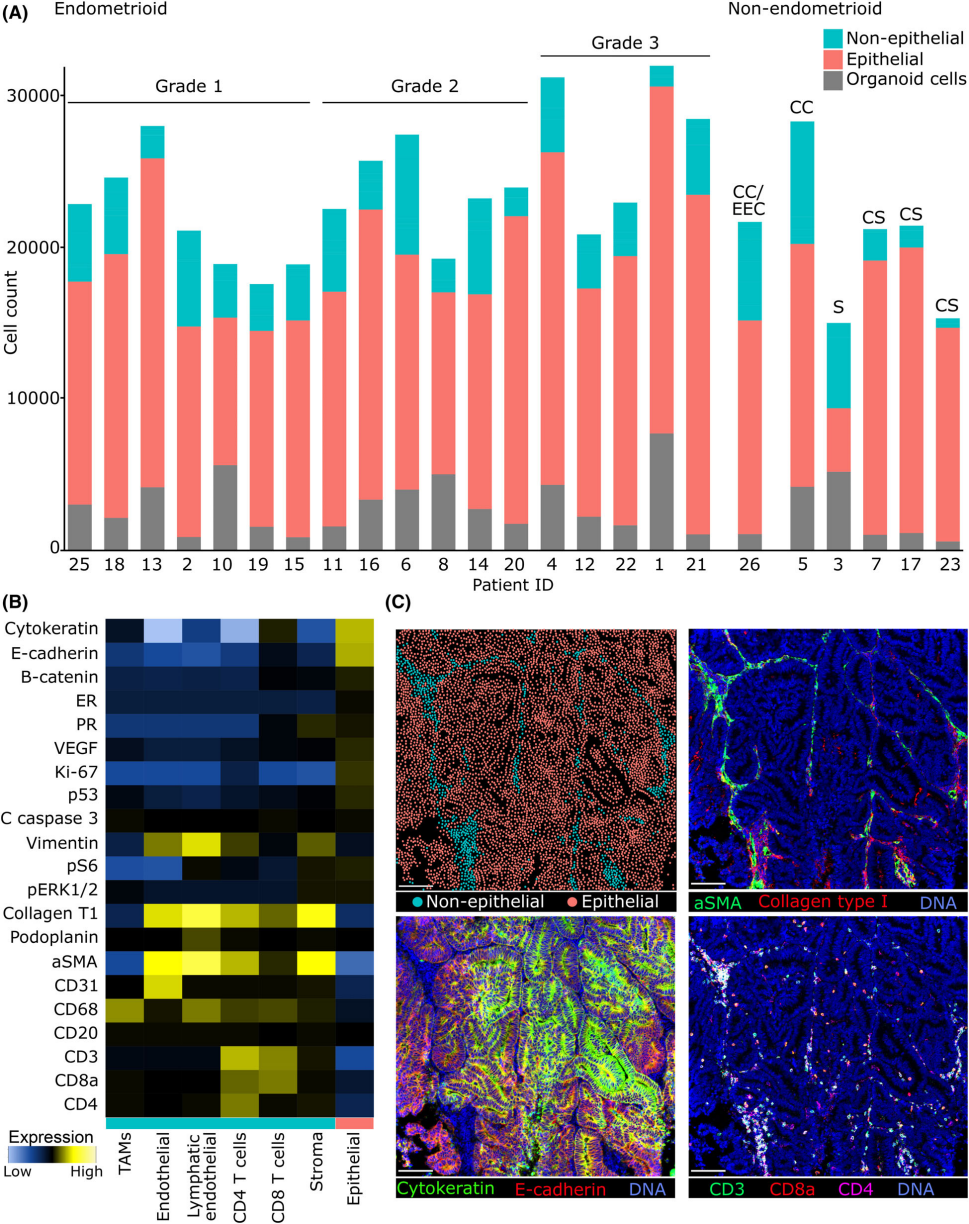
Cell count of epithelial and non‐epithelial cells reveal most samples are enriched for epithelial cell. (A) Cell count of tumor biopsy cells (epithelial and non‐epithelial) and corresponding organoid cells per patient sample. (B) Marker enrichment modeling (MEM) heatmap showing the relative marker expression of epithelial, stromal and immune markers of the different cell types in the tumor biopsies, used to identify epithelial cells. (C) Example IMC images showing stromal marker expression (upper right), epithelial marker expression (lower left) and immune marker expression (lower right), used to identify epithelial cells. The upper left image shows dots representing single cells with similar spatial relations as in the tissue image. The colors represent if the cell is annotated as epithelial or non‐epithelial. Scale bar in white indicate 100 μm. aSMA, αSMA; B‐catenin, β‐catenin; C caspase 3, cleaved caspase 3; Collagen T1, Collagen type 1; CS, carcinosarcoma; EEC, endometrioid endometrial cancer; ER, estrogen receptor; PR, progesterone receptor; S, serous; TAMs, tumor associated macrophages.

Single cells were segmented from IMC images as described in the material and methods. In total, 554 669 single cells were segmented with a median of 22 778 cells per patient (range 15 055 to 32 341 cells) including organoid cells. Cells were clustered by unsupervised phenograph clustering and the clusters were annotated into non‐epithelial and epithelial cells (Fig. 1A,B) as described in the material and methods section. The epithelial and non‐epithelial cells were visualized by their spatial location in the tissue image to validate the cellular annotation (Fig. 1C).

**Fig. 2:**
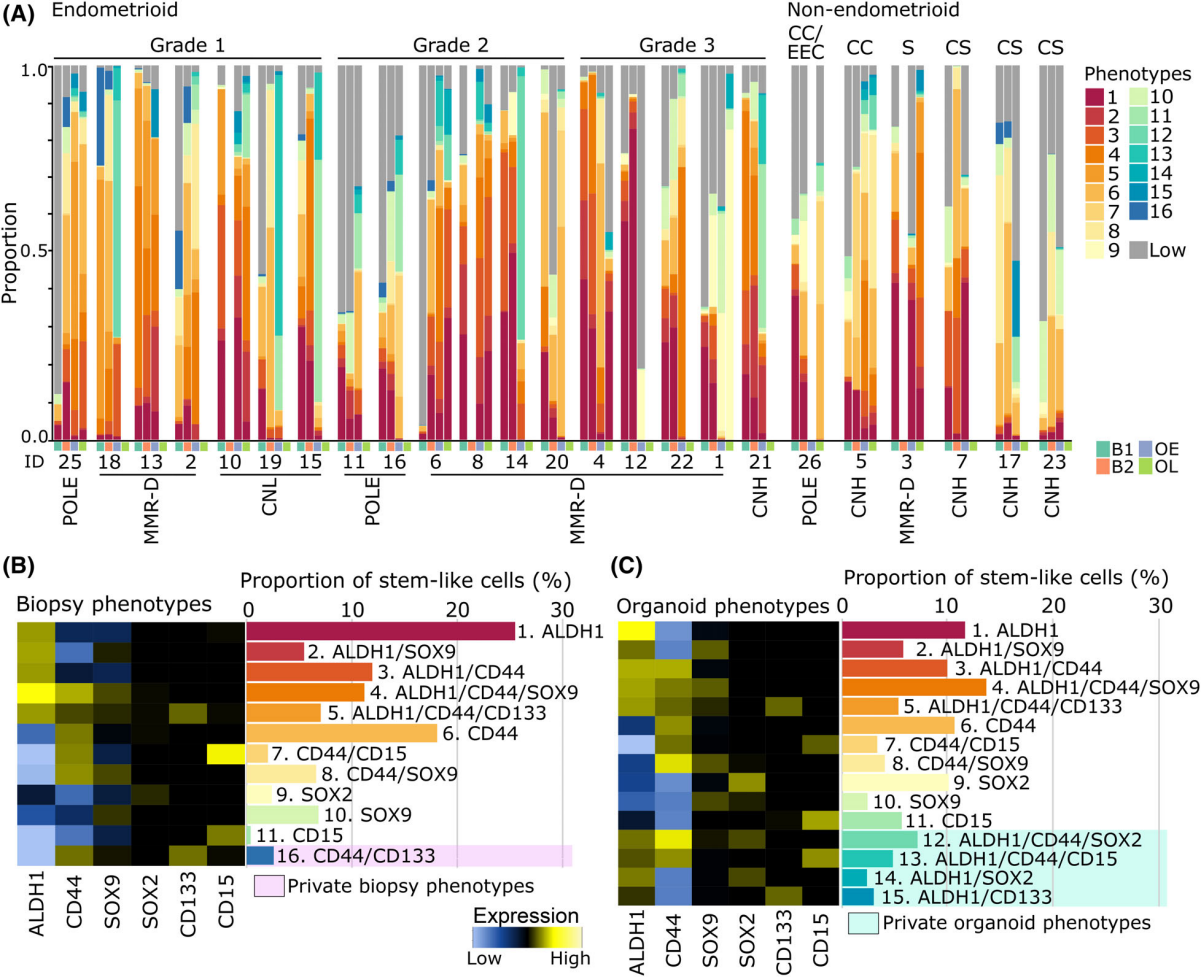
Characterization of cancer stem cell phenotypes reveals heterogeneous expression between patients and organoids. (A) Proportional bar plot showing the distribution of the cancer stem cell phenotypes and cells not expressing cancer stem cell markers (Low) in each tumor biopsy and organoid sample ordered by grade and histologic subtype. (B) MEM heatmap showing the relative expression intensity of stemness‐associated markers for the phenotypes identified in tumor biopsies. Yellow indicates higher expression and blue lower expression. The bar plot to the right shows the proportion of each phenotype from the total number of cells expressing cancer stem cell markers. Phenotypes private for tumor biopsies are highlighted in pink. (C) Marker enrichment modeling (MEM) heatmap with relative expression intensity of stemness markers in phenotypes identified in organoids. Phenotypes private to organoids are highlighted in blue. The bar plot to the right shows the proportion of each phenotype from the total number of organoid cells expressing cancer stem cell markers. B1, Biopsy 1; B2, Biopsy 2; CC, clear cell; CNH, copy number high; CNL, copy number low; CS, carcinosarcoma; EEC, endometrioid endometrial cancer; MMR‐D, mismatch repair‐deficient; OE, Organoid early; OL, Organoid late; POLE, POLE ultramutated; S, serous.

To characterize the expression patterns of proposed stem cell markers, epithelial cells from the biopsies and organoids were clustered based on the expression levels of CD44, ALDH1, SOX9, SOX2, CD133 and CD15. To validate the method, epithelial phenotypes were defined based on well‐known endometrial cancer markers [31]. As expected, the phenotypes were in general grade and subtype‐specific (Fig. S1a) and cells with higher expression of ER, PR, E‐cadherin, cytokeratin and β‐catenin were more prevalent in low‐grade tumors. To investigate cancer stem cell phenotypes, biopsy cells and organoid cells were clustered separately to increase the likelihood of detecting rare phenotypes. Overall, 74% (individual samples range 4–98%) of epithelial biopsy cells and 89% (individual samples range 19–99%) of organoid cells expressed one or more stemness‐associated markers. The distribution of the cancer stem cell phenotypes revealed considerable heterogeneity within the tumor biopsies, between patients and between the tumor biopsies and corresponding organoids (Fig. 2A). In total, 16 phenotypes were identified, where 11 overlapped between the biopsies and organoids (Fig. 2B,C). ALDH1 and CD44 were widely expressed and only three phenotypes did not express either ALDH1 or CD44, all three expressing only one marker (SOX2, SOX9 and CD15; Fig. 2B,C).

### 3.2 CD15 expression is enriched in endometrial cancer organoids

We hypothesized that specific stem cell markers essential in endometrial cancer would be enriched in the organoids. In organoid models from endometrioid tumors, cells expressing ALDH1 or SOX9 alone and cells not expressing any stemness markers (low) were less prevalent while cells expressing only CD15 were significantly more prevalent (Table 1). Only a few primary tumors had positive CD15 expression. In patient 19, scarce expression of CD15 was seen in the tumor biopsy, but CD15 was widely expressed in the organoid. Evident epithelial CD15 expression was observed in the tumor biopsy of patient 22 (indicated by white arrows, Fig. S2). Expression of CD15 was observed in two organoid glands of the corresponding organoid (Fig. S2, Organoid 22). We observed one non‐endometrioid tumor biopsy with a high proportion of infiltrating CD15 expressing cells. Minimal CD15 expression was seen in the corresponding organoid (patient and organoid 5; Fig. S2). None of the remaining stem cell markers showed differential expression between tumors and organoids, neither in endometrioid nor in non‐endometrioid tumors (Table S3).

**Table 1:** Mean percentage of stem‐like phenotypes in endometrioid biopsies and organoids from total epithelial cells per sample.

Phenotype	Sample	*N* of sample with phenotype	Mean % of phenotype per sample [range]	*P*‐value*
ALDH1	Biopsy	18/18	21.24 [1.31–69.83]	<0.001
	Organoid	15/18	6.13 [0–25.97]	
ALDH1/SOX9	Biopsy	17/18	4.71 [0–18.73]	0.203
	Organoid	15/18	4.47 [0–22.63]	
ALDH1/CD44	Biopsy	18/18	9.63 [0.50–34.47]	0.192
	Organoid	17/18	7.39 [0–28.96]	
ALDH1/CD44/SOX9	Biopsy	18/18	8.61 [0.17–38.55]	0.815
	Organoid	15/18	11.76 [0–71.86]	
ALDH1/CD44/CD133	Biopsy	18/18	6.83 [0.01–36.75]	0.161
	Organoid	14/18	3.86 [0–26.67]	
CD44	Biopsy	17/18	11.72 [0–34.76]	0.406
	Organoid	18/18	10.09 [0.05–55.91]	
CD44/CD15	Biopsy	13/18	0.14 [0–1.29]	0.406
	Organoid	11/18	3.53 [0–32.34]	
CD44/SOX9	Biopsy	14/18	3.67 [0–11.68]	0.719
	Organoid	14/18	3.67 [0–33.29]	
SOX2	Biopsy	18/18	1.41 [0.005–16.49]	0.079
	Organoid	8/18	4.87 [0–68.04]	
SOX9	Biopsy	18/18	4.13 [0.08–16.50]	<0.001
	Organoid	14/18	1.20 [0–11.37]	
CD15	Biopsy	14/18	0.11 [0–0.84]	<0.001
	Organoid	17/18	10.61 [0–64.69]	
ALDH1/CD44/SOX2	Biopsy	0/18	0	–
	Organoid	11/18	8.24 [0–70.36]	
ALDH1/CD44/CD15	Biopsy	0/18	0	–
	Organoid	16/18	8.44 [0–69.61]	
ALDH1/SOX2	Biopsy	0/18	0	–
	Organoid	14/18	1.28 [0–7.57]	
ALDH1/CD133	Biopsy	0/18	0	–
	Organoid	13/18	1.68 [0–12.87]	
CD44/CD133	Biopsy	16/18	2.23 [0–15.55]	–
	Organoid	0/18	0	
Low	Biopsy	18/18	28.96 [1.02–65.81]	0.020
	Organoid	18/18	12.77 [0.14–80.97]	

* Mann–Whitney U test.

### 3.3 ALDH1 and CD44 expression associate with histologic grade

**Fig. 3:**
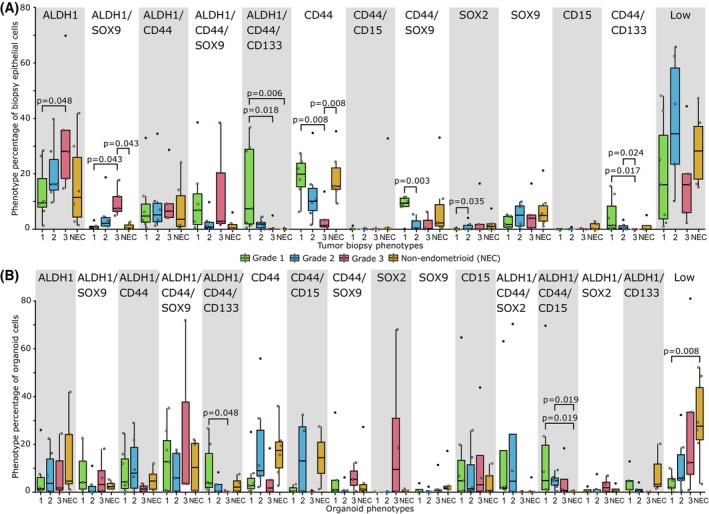
Specific cancer stem cell phenotypes in tumor biopsies associate with histologic grade. (A) Boxplot showing the proportion of the tumor biopsy cancer stem cell phenotypes of the total number of epithelial cells compared by grade and non‐endometrioid tumors. (B) Boxplot showing the proportion of the organoid cancer stem cell phenotypes of the total number of organoid cells compared by grade and non‐endometrioid organoids. Groups were compared using Mann–Whitney U test. NEC, Non endometrioid tumors.

Next, we investigated if specific cancer stem cell phenotypes were associated with histologic grade or subtype. ALDH1 and ALDH1/SOX9 expressing cells were significantly more prevalent in grade 3 tumor biopsies (Fig. 3A). Four phenotypes characterized by expression of CD44 and CD133 (ALDH1/CD44/CD133, CD44, CD44/SOX9 and CD44/CD133) were more prevalent in grade 1 tumor biopsies (Fig. 3A). In the organoids, the proportion of ALDH1/CD44/CD133 and ALDH1/CD44/CD15 cells were higher in grade 1 organoids (Fig. 3B). Notably, the proportion of cells not expressing any stem markers (low) was significantly higher in non‐endometrioid organoids (Fig. 3B). To investigate if individual stem markers alone were associated with grade or subtype, we analyzed the distribution of single marker positive cells (Fig. S3a). In the tumor biopsies, cells expressing CD44 and cells expressing CD133 were more prevalent in grade 1 tumors. In the organoids, cells expressing ALDH1 and cells expressing CD133 were more prevalent in grade 1 tumors (Fig. S3b).

### 3.4 Spatial interaction analysis of cancer stem cell phenotypes reveals less contact between CD44 and ALDH1 expressing cells

**Fig. 4:**
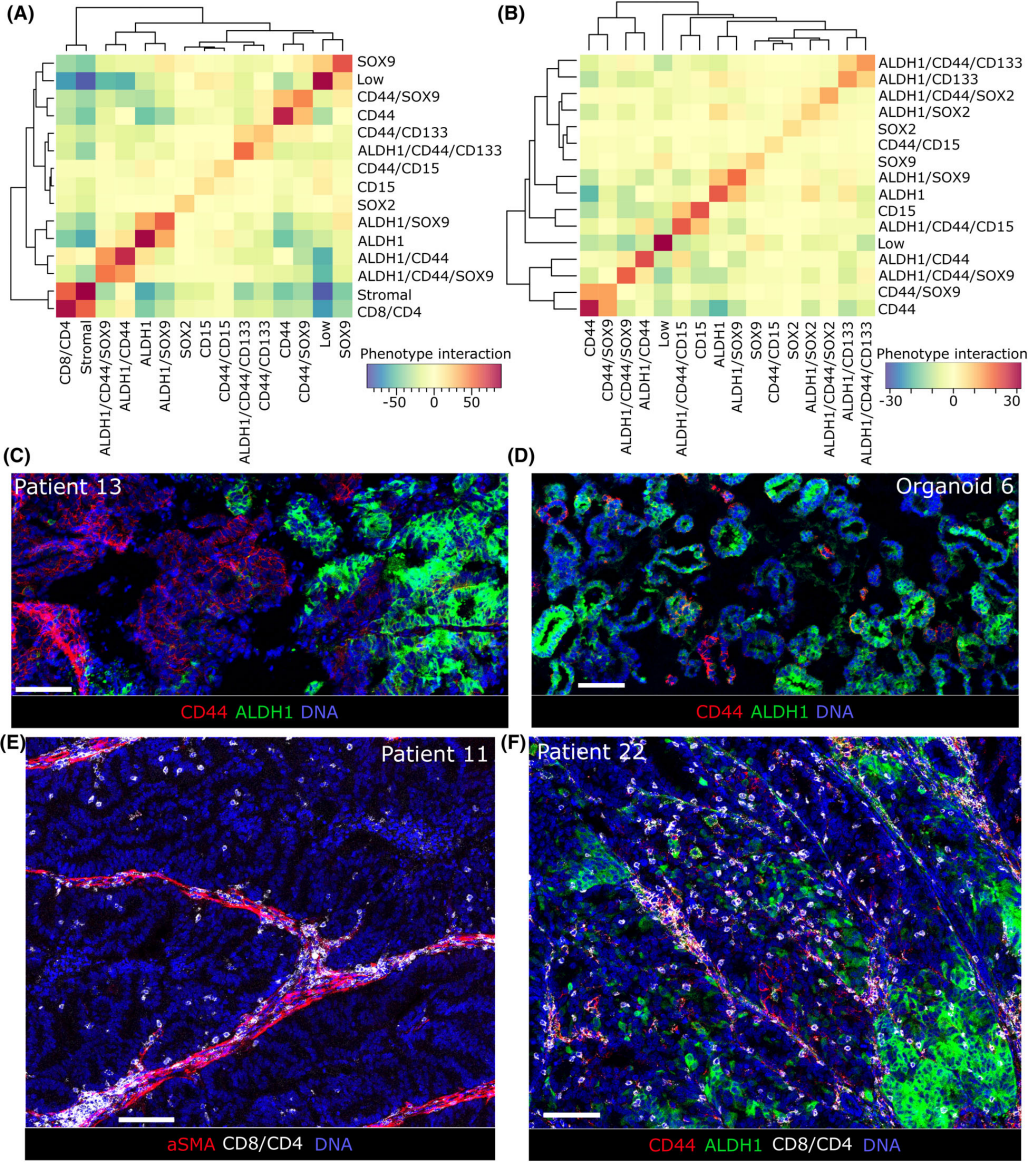
Interaction analysis of cancer stem cell phenotypes reveals no interaction between CD44 expressing cells and ALDH1 expressing cells. (A) Heatmap showing cumulative significant interaction or no interaction between tumor biopsies phenotypes expressing cancer stem cell markers. (B) Heatmap of cumulative significant interaction or no interaction between organoid phenotypes expressing cancer stem cell markers. Red indicates interaction and blue indicates avoidance, while yellow indicates no significant interaction between phenotypes. (C) Example Imaging mass cytometry (IMC) image of avoidance between CD44 (red) and ALDH1 (green) in the tumor biopsy of patient 13. (D) Example image of avoidance between CD44 (red) and ALDH1 (green) in the organoid from patient 6. (E) Example image of T cell location (CD8/CD4 in white) in relation to αSMA (red) expressing stromal cells. (F) Example image of T cell location (CD8/CD4 in white) in relation to ALDH1 (green) and CD44 (red) expressing cells. DNA shown in blue. Scale bar in white indicate 100 μm.

Interaction analyses of the cancer stem cell phenotypes, T cells and stromal cells revealed that phenotypes with similar marker expression tended to more closely interact, such as ALDH1/CD44 with ALDH1/CD44/SOX9, ALDH1 with ALDH1/SOX9 and CD44 with CD44/SOX9 (Fig. 4A,B). Interestingly, cells expressing only ALDH1 did not interact with cells expressing only CD44 in both biopsies and organoids (Fig. 4A,B, and example image from tumor biopsy Fig. 4C, and organoid Fig. 4D). In the tumor biopsies, a closer interaction was seen between T cells and stromal cells (Fig. 4A and example image from tumor biopsy Fig. 4E). T cells and stromal cells showed no clear interaction with any cancer stem cell phenotype, nor with cells not expressing any cancer stem cell markers (Fig. 4A, example image from tumor biopsy Fig. 4F).

### 3.5 High expression of CD44 associates with low‐grade disease and better outcome

**Fig. 5:**
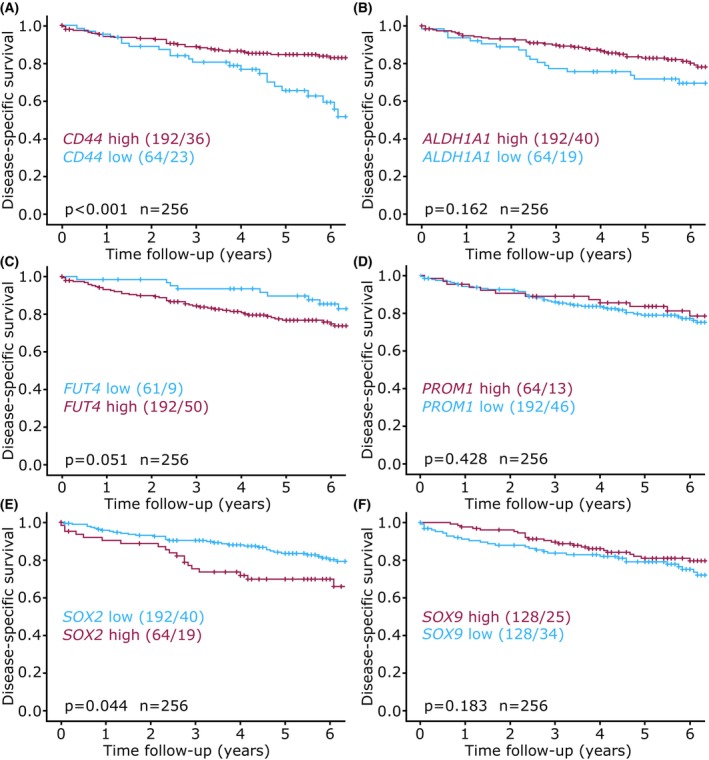
Disease‐specific survival of patient groups stratified by gene expression of *CD44*, *ALDH1A1*, *FUT4*, *PROM1*, *SOX2* and *SOX9* Kaplan–Meier survival curve of high and low gene expression groups of (A) *CD44*, (B) *ALDH1A1*, (C) *FUT4* (CD15), (D) *PROM1* (CD133), (E) *SOX2* and (F) *SOX9*. Kaplan–Meier survival curves presented with number of patients in each group and number of events in parentheses (patients/events). *P*‐values from Mantel–Cox log‐rank test.

Gene expression data for the cancer stem cell markers was available from a larger patient cohort (*n* = 256). To investigate if any of the selected stem cell markers were widely expressed in aggressive disease, expression level data was analyzed in association to prognosis. Among the selected markers, high gene expression of *CD44* was associated with better disease‐specific survival (Log‐rank *P* < 0.001, Fig. 5A) and better recurrence‐free survival (Log rank *P* = 0.003, data not shown). In contrast, high expression of *SOX2* was associated with worse prognosis (Log‐rank *P* = 0.044; Fig. 5E). No significant association was observed for the remaining cancer stem cell markers (Fig. 5). *CD44* results were validated in an external gene expression dataset from TCGA (Fig. S4). Both disease‐specific and progression‐free survival was significantly higher for endometrial cancer patients with high expression of *CD44* (Fig. S4a,b).

**Fig. 6:**
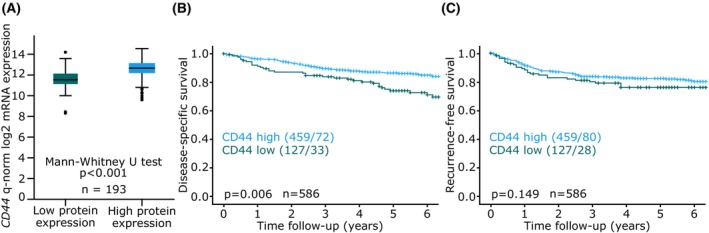
High expression of CD44 is associated with better patient outcome. (A) Boxplot showing the comparison of gene expression and protein expression of CD44 in overlapping cases between the mRNA and protein expression data (*n* = 193). (B) Kaplan–Meier survival curves of (B) disease‐specific survival and (C) recurrence‐free survival for patients with high or low protein expression of CD44. Kaplan–Meier survival curves presented with number of patients in each group and number of events in parentheses (patients/events). *P*‐values from Mantel–Cox log‐rank test.

The strong association between high *CD44* and better survival was further investigated at protein level to evaluate the robustness of this finding. Tumor tissue from 586 patients was stained for CD44 by IHC. Protein expression of CD44 significantly correlated with gene expression of *CD44*, in overlapping data from 193 patients (Mann–Whitney U test *P* < 0.001, Fig. 6A). High protein expression of CD44 associated with lower age at diagnosis, endometrioid subtype, lower FIGO stage, low histologic grade, CNL and MMR‐deficient molecular subgroups (Table S4). High protein expression of CD44 was associated with better disease‐specific survival (Log rank *P* = 0.006, Fig. 6B), but not significantly associated with recurrence‐free survival (Log rank *P* = 0.149, Fig. 6C).

## 4 Discussion

Cancer stem cells are linked to metastasis and poor patient outcome, and several markers of cancer stem cells have been proposed [11, 33]. We here investigated expression patterns of cancer stem cell markers reported from previous studies on endometrial cancer, and transcription factors ascribed with universal roles in stemness, to assess the relevance of these markers specifically in endometrial cancer.

Cancer stem cell phenotypes were defined based on expression of one or more of the included cancer stem cell markers in our antibody panel. The phenotypes were heterogeneous among different biopsies from individual patients, as well as between tumor biopsies and organoid samples. Most of the stem‐marker positive cells expressed CD44, ALDH1, SOX9 and CD133 either alone or in combination with other stem cell markers. Surprisingly, more than 70% of the epithelial cells segmented from the tumor biopsies expressed one or more proposed cancer stem cell markers. However, the fraction of defined stem cells varied between samples, from less than 5% to more than 90%. Previous studies report varied frequency of cancer stem cells, ranging from very low (<1%) to high (more than 90%) and depending on the selected stem marker [34, 35, 36, 37, 38, 39, 40, 41]. Similar findings are also reported for the cancer stem cell markers included in our study, where both range of expression and variability between cancer types are evident.

Few detailed studies characterizing the expression of endometrial cancer stem markers have been published, and most reports describe single marker expression. For CD44, 90% of endometrial epithelial cells [42] and 40–60% of tumors have been reported as positive [43]. Similarly, for ALDH1, around 40% of endometrial tumors expressed ALDH1 in more than 10% of epithelial cells [43], over 85% expressed CD133, including 61% of the tumors with expression in more than 10% of cancer cells [44]. Expression of CD44 and CD133 is also reported in normal endometrium [45, 46], and in post‐menopausal normal endometrium, where over 75% of epithelial cells show SOX9 expression [15]. The present study and previously reported data suggest that cancer stem cell markers are widely expressed in both normal and malignant endometrial tissue. This might indicate that endometrial tumors have a highly stemness stimulating microenvironment or that these markers have more general roles, not restricted to stemness. The variability in expression could also imply plastic expression of cancer stem cell markers, regulated by external stimuli [47]. Secretion of different signaling molecules from the tumor microenvironment could differ between patients and induce variable expression of stemness markers in the cancer stem cells [47]. We did not observe any significant interaction between stromal or immune cells and any of the cancer stem cell phenotypes. T cells showed strong interaction with stromal cells, but not with epithelial cells, indicating that none of the cancer stem cell phenotypes are distinctly immunogenic [48].

We hypothesized that stem cell markers could be enriched in organoids to allow cells to continuously grow in culture. However, we observed few differences between tumor biopsies and corresponding organoids and only CD15 expression was more prevalent in the patient‐derived organoids compared to the tumor biopsies. Expression of CD15 on cancer cells has been linked to worse survival and metastatic disease [49]; however, CD15 expression was not associated with grade in our study. Most low‐grade organoid samples had more expression of stem cell markers, compared to non‐endometrioid organoids where the fraction of the low phenotype (no detected stem marker) was significantly higher. Dissociation of tissue may upregulate CD15 and favor organoid formation [47], due to the cell adhesion function of CD15 [50, 51, 52]. This is supported by increased formation success for spheroids or organoids from normal tissue or well‐differentiated cancer cells [53, 54, 55, 56]. In line with this, our results could indicate that cell adhesion is equally important to stemness when low‐grade organoids are successfully established. The lack of differences in cancer stem cell phenotypes between non‐endometrioid tumor biopsies and corresponding organoids might indicate that alternative cancer stem cell markers not included in our panel or other mechanisms are involved in successful establishing of viable high‐grade and non‐endometrioid organoid cultures. Detailed functional studies are needed to pinpoint the role of CD15 in organoid formation from endometrial cancer.

Given the wide expression of the cancer stem cell markers, we also explored if any of these had prognostic value in endometrial cancer. ALDH1 and CD44 were expressed in 13 of the 16 identified cancer stem cell phenotypes and have previously been linked to patient outcome [43], implying a relevance to cancer in general. Data from the IMC analyses indicated that grade 3 tumor biopsies had a higher proportion of cells expressing ALDH1 alone. This is in line with multiple studies where high expression of ALDH1 is associated with poor prognosis [43, 57]. However, gene expression of *ALDH1A1* did not associate with prognosis in a larger patient cohort limiting its prognostic value. In contrast, we found that both gene expression levels and protein expression of CD44 associate with prognosis. This was also validated in an external gene expression dataset from TCGA, where high expression of *CD44* was significantly associated with better prognosis. The role of CD44 in cancer is unclear. Previous studies have reported high expression of CD44 to associate with worse outcome in several cancers [58, 59], including a smaller cohort of endometrial cancer patients (*n* = 113) [43]. This is in contrast to a report describing CD44 to associate with less aggressive disease in colorectal cancer [60]. Expression of CD44 has been reported to be lower in endometrial cancer than in normal tissue and loss of CD44 associated with lymphovascular space invasion [46]. This is in concordance with our findings where high protein expression (IHC) of CD44 is associated with endometrioid subtype, low‐grade and better disease‐specific survival. We also observed significantly less physical interaction between cells expressing ALDH1 only and cells expressing CD44 only. This could indicate that ALDH1‐only cells represent a more aggressive phenotype often linked to higher grade that is physically separated from the more differentiated CD44 expressing cells. However, it should be noted that the patients in this study were selected based on the successful establishment of corresponding organoid models, giving an overrepresentation of low‐stage primary tumors.

## 5 Conclusions

In summary, we have identified epithelial phenotypes specific to tumor grade and subtype. High expression of suggested endometrial cancer stem cell markers is not indicative of aggressive disease, notably high expression of CD44 is linked to better clinical outcome. As several of the cancer stem cell markers included in our study have alternative roles not restricted to cancer stem cells, this study highlights the need for further research on reported cancer stem cell markers in endometrial cancer to validate the precise role and function of these cells.

## Conflict of interest

The authors declare no conflict of interest.

## Author contributions

HEL, ISH, LAA and CK conceived the experiment. HEL conducted the experiments. HEL and RMG processed the data and performed bioinformatic analyses. KW contributed to the collection of samples and clinical data. HEL, MEH, HFB and CK contributed to the interpretation of the results. HEL, MEH and CK wrote the manuscript. CK supervised the project. HEL and CK have verified the underlying data. All authors provided feedback on the research and revision of the manuscript. All authors read and approved the manuscript.

## Peer review

The peer review history for this article is available at https://www.webofscience.com/api/gateway/wos/peer‐review/10.1002/1878‐0261.13815.

## Supporting information


**Fig. S1.** Identification of grade specific epithelial phenotypes in tumor biopsies and corresponding organoids. (a) Proportional bar plot showing the distribution of the epithelial phenotypes in each sample, ordered by grade and histologic type. Each bar represents one sample of biopsy 1, biopsy 2, early organoid culture or late organoid culture. (b) MEM heatmap showing the relative marker expression intensity of each epithelial phenotype. Yellow indicates higher expression and blue lower expression. The dendrogram on the left of the heatmap was created by hierarchical clustering of the epithelial phenotypes. The colors on the dendrogram indicate the main phenotype cluster C1 (magenta) or C2 (teal). (c) UMAPs of tumor biopsy epithelial cells and all organoid cells colored by the main phenotype clusters for all samples combined and for each grade and non‐endometrioid samples. aSMA, αSMA; B‐catenin, β‐catenin; B1, Biopsy 1; B2, Biopsy 2; CC, clear cell; C caspase 3, cleaved caspase 3; CNH, copy number high; CNL, copy number low; Collagen T1, collagen type 1; CS, carcinosarcoma; EEC, endometrioid endometrial cancer; MMR‐D, mismatch repair‐deficient; OE, Organoid early; OL, Organoid late; POLE, POLE ultra mutated; S, serous.

**Fig. S2.** CD15 expression in tumor biopsies and corresponding organoids reveals heterogeneous expression. Example IMC images of tumor biopsy and corresponding organoid of patient 19 (top panels), patient 22 (middle panels) and patient 5 (bottom panels). Cell DNA is shown in blue pseudo color, the epithelial marker E‐cadherin in green and CD15 in red. White arrows indicate clear epithelial CD15 expression in the tumor biopsy of patient 22. Scale bar in white indicate 100 μm.

**Fig. S3.** Total proportion of single marker stem cell populations of the total epithelial cell number in each sample. (a) Boxplots showing the proportion of single marker positive cells of the total tumor biopsy epithelial cells for each sample by grade and histologic type. (b) Boxplots showing the proportion of single marker positive cells of the total organoid cell number for each sample by grade and histologic type. The proportion of the low phenotype in the tumor biopsies is given in Fig. 3a and for organoids in 3b.

**Fig. S4.** Gene expression of *CD44* from TCGA gene expression data shows that high expression of *CD44* is associated with favorable disease‐specific and progression‐free survival. (a) Kaplan‐Meier survival curves showing disease‐specific survival and (b) progression‐free survival for patients with high or low gene expression of CD44. Kaplan Meier survival curves presented with number of patients in each group and number of events in parentheses (patients/events). *P*‐values from Mantel Cox log‐rank test.

**Table S1.** Clinical characteristics of IMC patient cohort.

**Table S2.** Panel of metal tagged antibodies for imaging mass cytometry.

**Table S3.** Mean percentage of stem‐like phenotypes in non‐endometrioid biopsies and organoids from total epithelial cells per sample.

**Table S4.** CD44 expression associates with clinicopathological variables.

## Data Availability

The microarray gene expression data is publicly available at ArrayExpress (accession number E‐MTAB‐2532). The IMC data is available from the corresponding author upon reasonable request. The TCGA mRNA dataset is publicly available at cbioportal.org (https://www.cbioportal.org/study/summary?id=ucec_tcga_pan_can_atlas_2018).
