# Supplementary figures and images for: Multiplex single‐cell profiling of putative cancer stem cell markers ALDH1, SOX9, SOX2, CD44, CD133 and CD15 in endometrial cancer
Source: Mol Oncol. 2025 Jan 31;19(6):1651–67. doi: 10.1002/1878-0261.13815 (PMC12161474; doi:10.1002/1878-0261.13815)

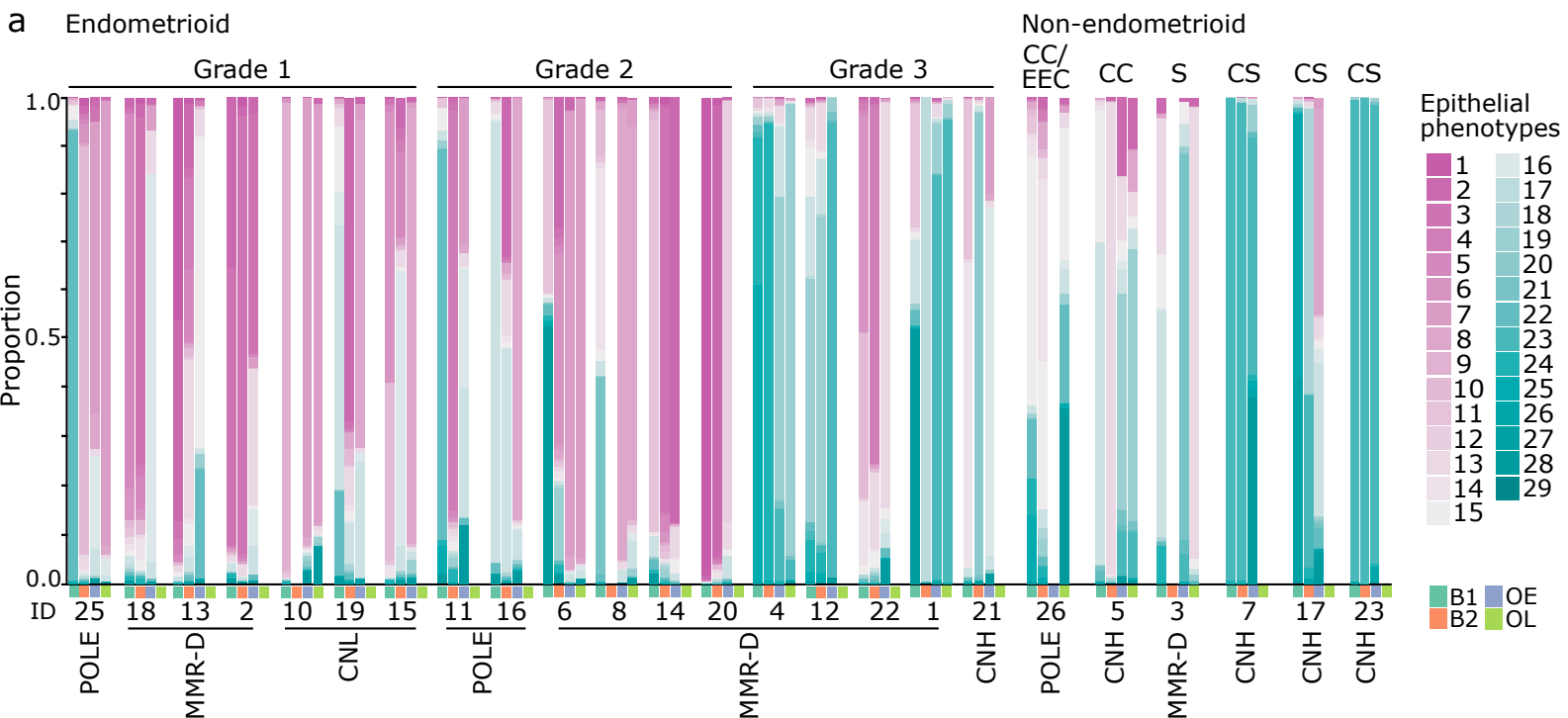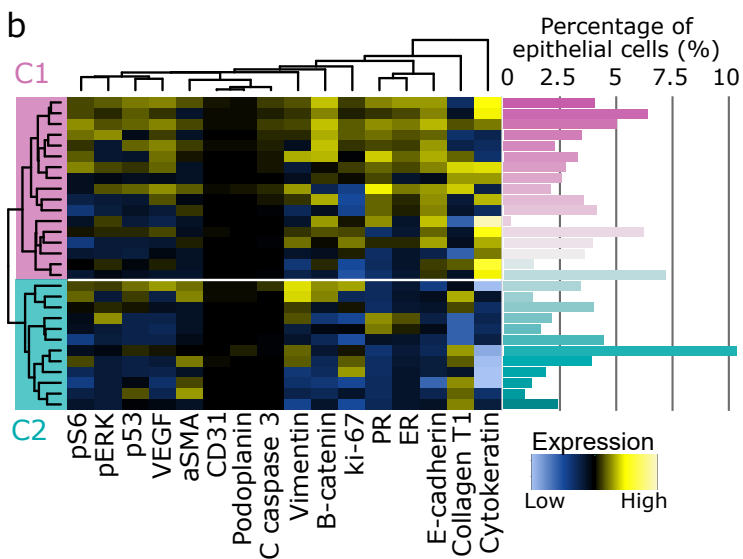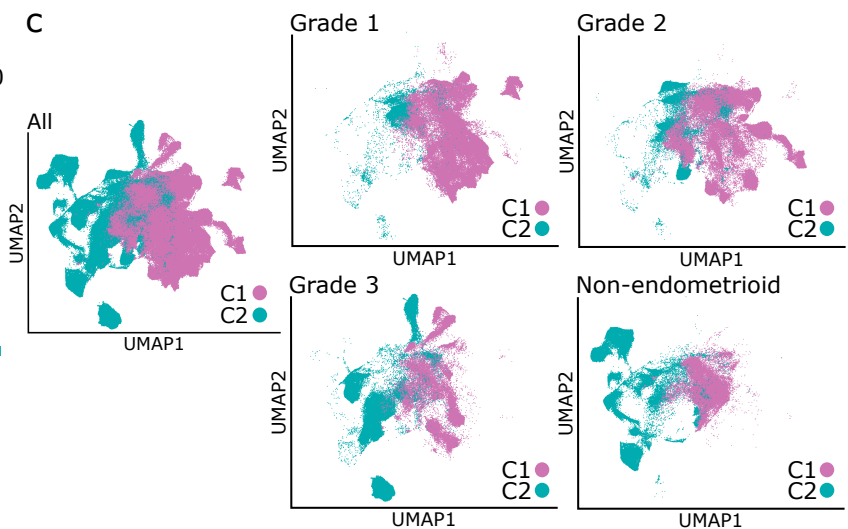

Supplement: Supplementary file 1 — Fig. S1. Identification of grade specific epithelial phenotypes in tumor biopsies and corresponding organoids. (a) Proportional bar plot showing the distribution of the epithelial phenotypes in each sample, ordered by grade and histologic type. Each bar represents one sample of biopsy 1, biopsy 2, early organoid culture or late organoid culture. (b) MEM heatmap showing the relative marker expression intensity of each epithelial phenotype. Yellow indicates higher expression and blue lower expression. The dendrogram on the left of the heatmap was created by hierarchical clustering of the epithelial phenotypes. The colors on the dendrogram indicate the main phenotype cluster C1 (magenta) or C2 (teal). (c) UMAPs of tumor biopsy epithelial cells and all organoid cells colored by the main phenotype clusters for all samples combined and for each grade and non‐endometrioid samples. aSMA, αSMA; B‐catenin, β‐catenin; B1, Biopsy 1; B2, Biopsy 2; CC, clear cell; C caspase 3, cleaved caspase 3; CNH, copy number high; CNL, copy number low; Collagen T1, collagen type 1; CS, carcinosarcoma; EEC, endometrioid endometrial cancer; MMR‐D, mismatch repair‐deficient; OE, Organoid early; OL, Organoid late; POLE, POLE ultra mutated; S, serous. [file MOL2-19-1651-s002.pdf]

Patient 19

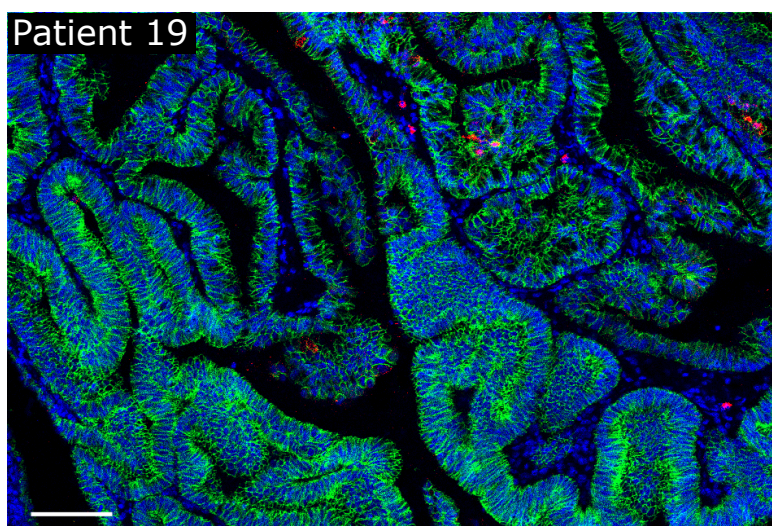

Organoid 19

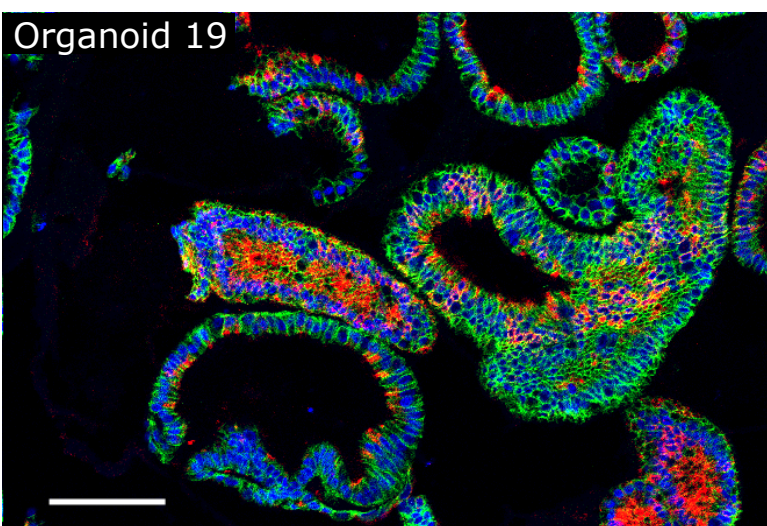

Patient 22

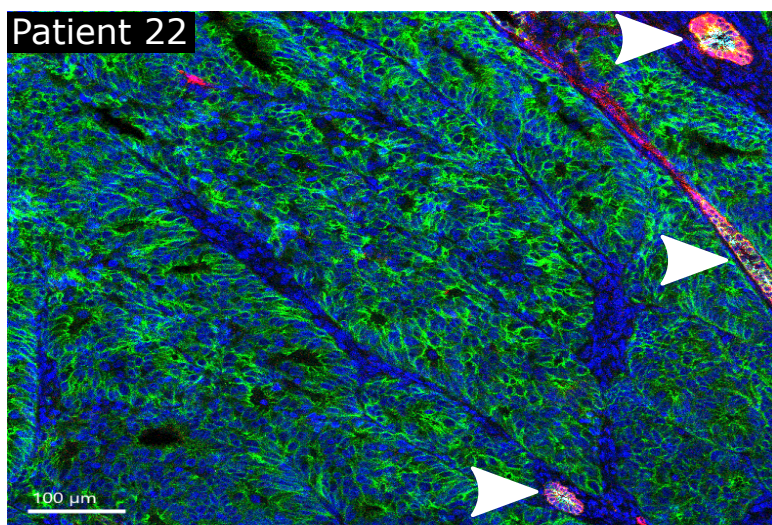

Organoid 22

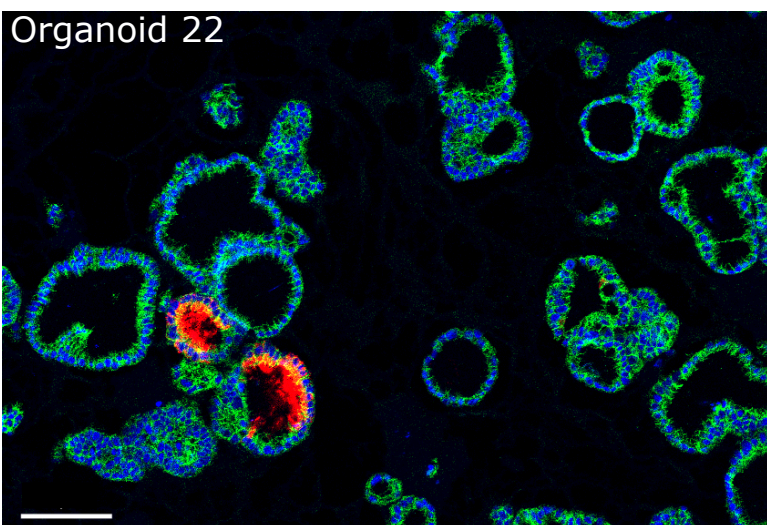

Patient 5

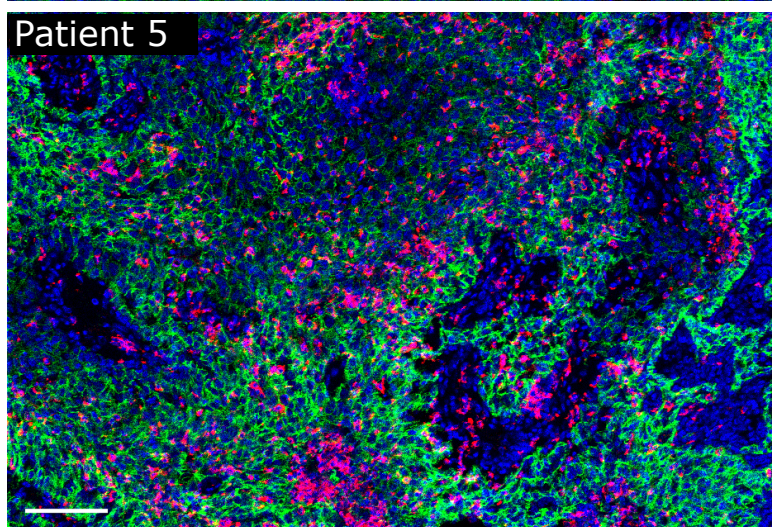

Organoid 5

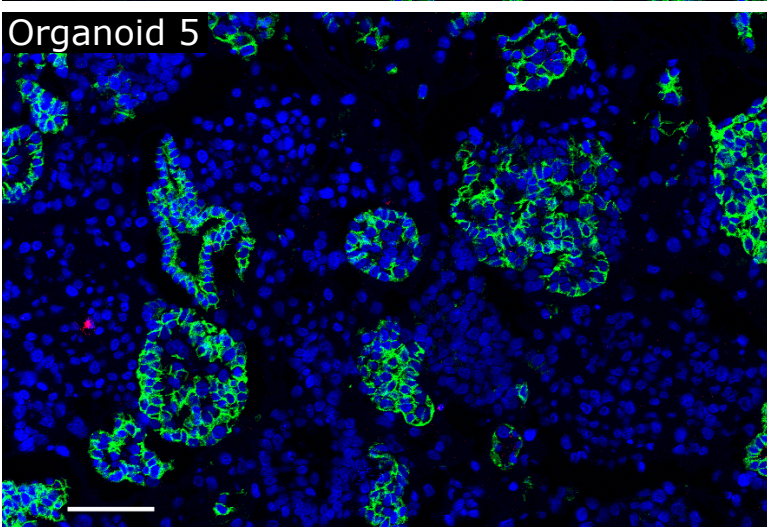

CD15

E-cadherin

DNA

Supplement: Supplementary file 2 — Fig. S2. CD15 expression in tumor biopsies and corresponding organoids reveals heterogeneous expression. Example IMC images of tumor biopsy and corresponding organoid of patient 19 (top panels), patient 22 (middle panels) and patient 5 (bottom panels). Cell DNA is shown in blue pseudo color, the epithelial marker E‐cadherin in green and CD15 in red. White arrows indicate clear epithelial CD15 expression in the tumor biopsy of patient 22. Scale bar in white indicate 100 μm. [file MOL2-19-1651-s007.pdf]

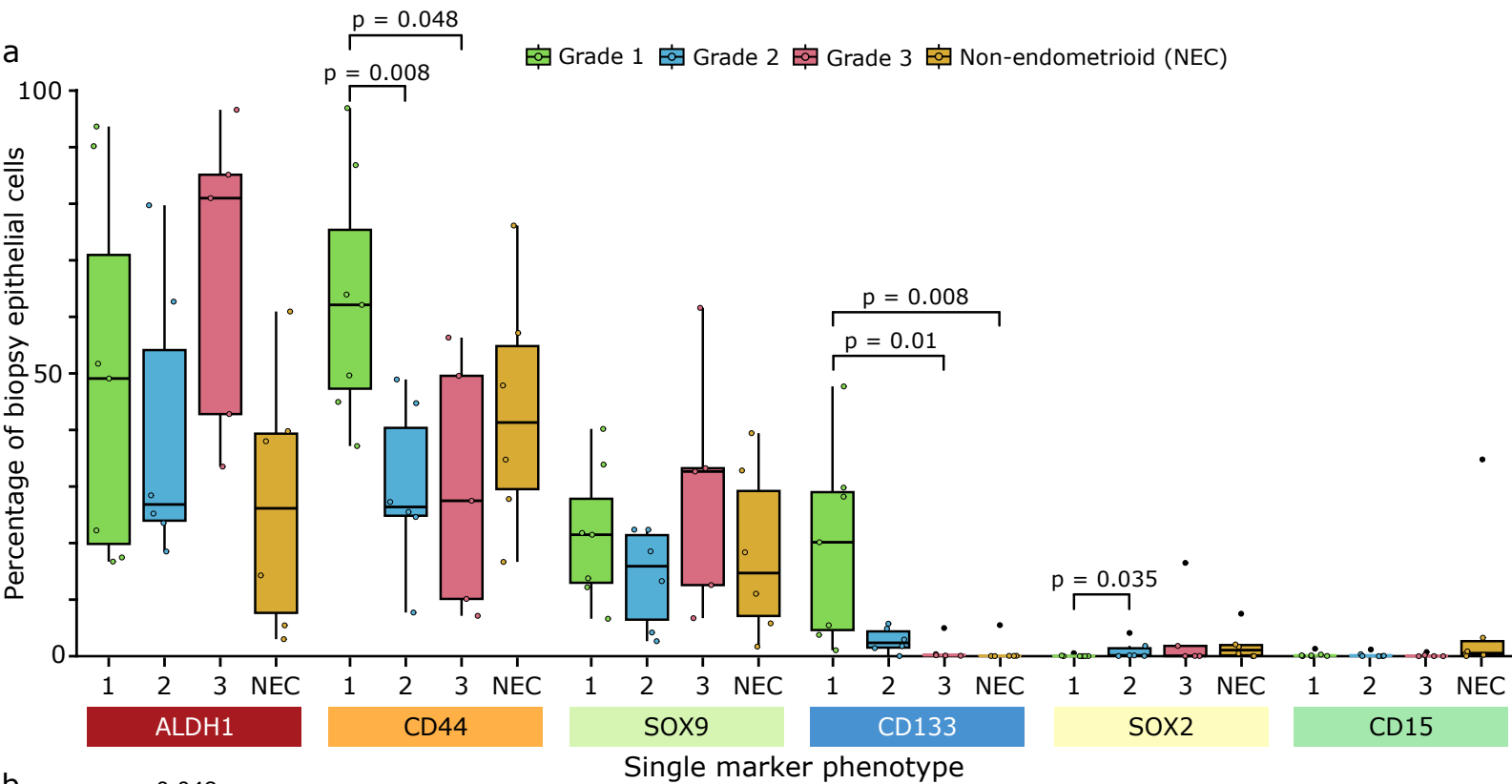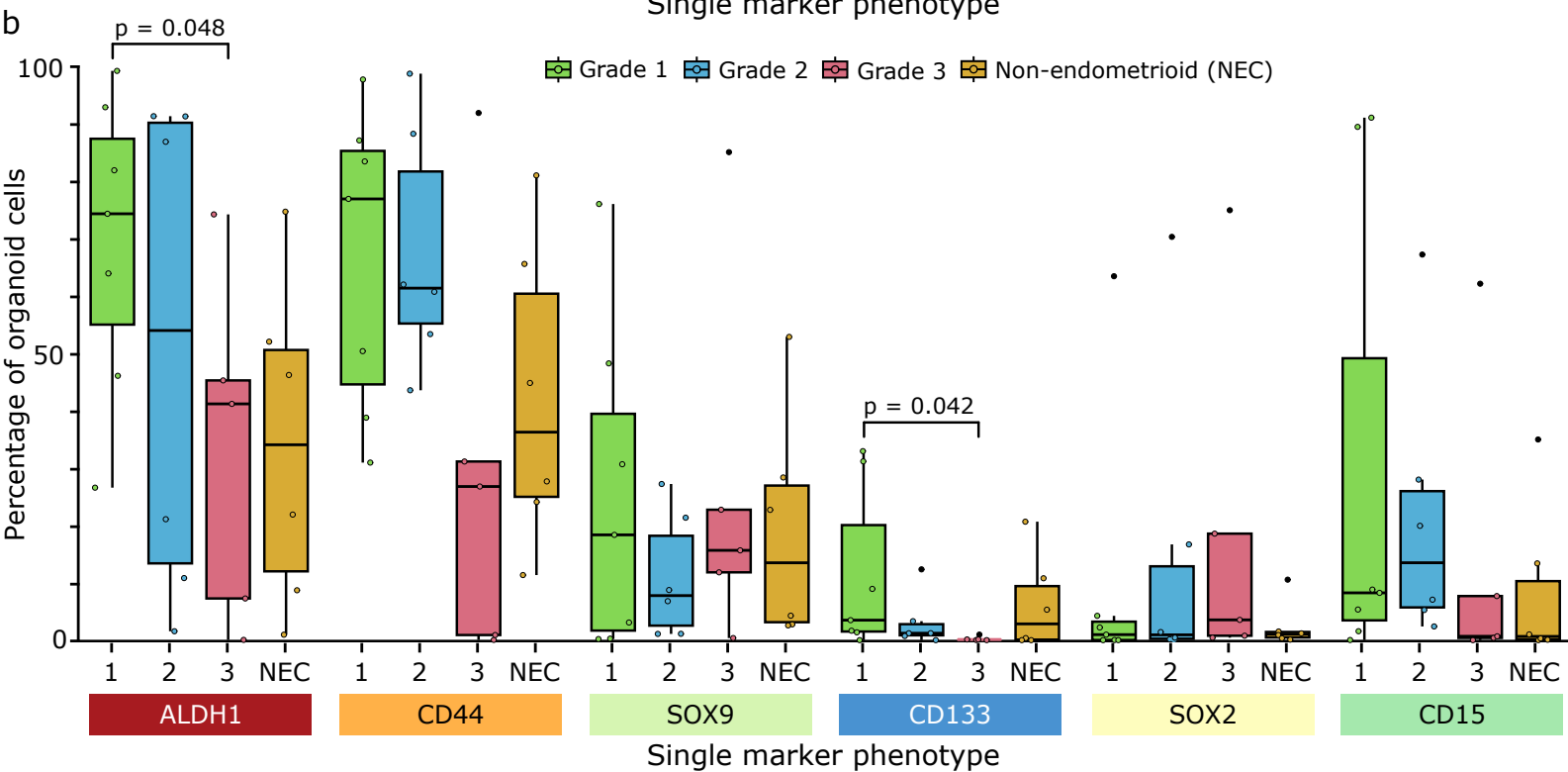

Supplement: Supplementary file 3 — Fig. S3. Total proportion of single marker stem cell populations of the total epithelial cell number in each sample. (a) Boxplots showing the proportion of single marker positive cells of the total tumor biopsy epithelial cells for each sample by grade and histologic type. (b) Boxplots showing the proportion of single marker positive cells of the total organoid cell number for each sample by grade and histologic type. The proportion of the low phenotype in the tumor biopsies is given in Fig. 3a and for organoids in 3b. [file MOL2-19-1651-s008.pdf]

# TCGA mRNA data of endometrial cancers

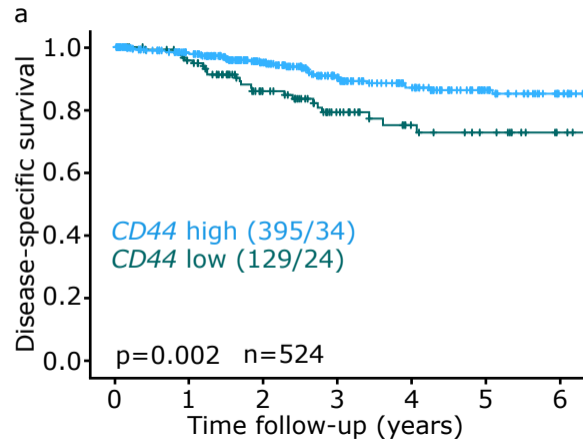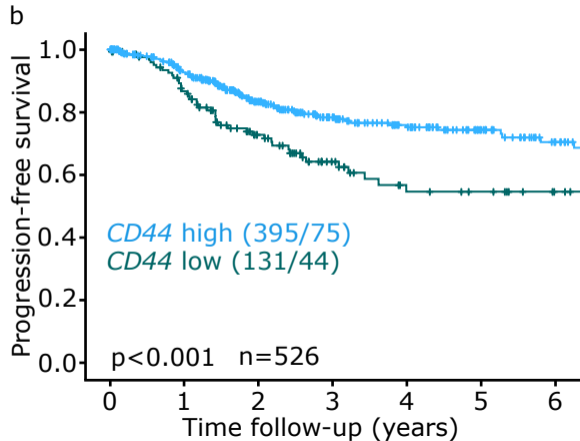

Supplement: Supplementary file 4 — Fig. S4. Gene expression of CD44 from TCGA gene expression data shows that high expression of CD44 is associated with favorable disease‐specific and progression‐free survival. (a) Kaplan‐Meier survival curves showing disease‐specific survival and (b) progression‐free survival for patients with high or low gene expression of CD44. Kaplan Meier survival curves presented with number of patients in each group and number of events in parentheses (patients/events). P‐values from Mantel Cox log‐rank test. [file MOL2-19-1651-s003.pdf]
