# Supplementary material for: Multiplex single‐cell profiling of putative cancer stem cell markers ALDH1, SOX9, SOX2, CD44, CD133 and CD15 in endometrial cancer
Source: Mol Oncol. 2025 Jan 31;19(6):1651–67. doi: 10.1002/1878-0261.13815 (PMC12161474; doi:10.1002/1878-0261.13815)
Supplement: Supplementary file 5 — Table S1. Clinical characteristics of IMC patient cohort. [file MOL2-19-1651-s005.docx]

**Supplementary table 1** Clinical characteristics of IMC patient cohort

| Characteristic | Patient cohort, n=24 n(%) |
| --- | --- |
| Age (years) |  |
| <66 | 14 (58) |
| ≥66 | 10 (42) |
| Histologic type |  |
| Endometrioid | 18 (75) |
| Non-endometrioid | 6 (25) |
| FIGO stage |  |
| I | 20 (83) |
| II | 2 (8) |
| III | 1 (4) |
| IV | 1 (4) |
| Histologic Grade* |  |
| I | 7 (39) |
| II | 6 (33) |
| III | 5 (28) |
| * Endometrioid only | |
