# Supplementary material for: Multiplex single‐cell profiling of putative cancer stem cell markers ALDH1, SOX9, SOX2, CD44, CD133 and CD15 in endometrial cancer
Source: Mol Oncol. 2025 Jan 31;19(6):1651–67. doi: 10.1002/1878-0261.13815 (PMC12161474; doi:10.1002/1878-0261.13815)
Supplement: Supplementary file 6 — Table S2. Panel of metal tagged antibodies for imaging mass cytometry. [file MOL2-19-1651-s006.docx]

**Supplementary table 2** Panel of metal tagged antibodies for imaging mass cytometry

| **Expressed in cell type** | **Tag** | **Target** | **Clone** | **Dilution (µg/ml)** | **Vendor** | **Cat#** | **RRID** |
| --- | --- | --- | --- | --- | --- | --- | --- |
| Epithelial | 148Nd | Pan-cytokeratin | AE1/AE3 | 6.67 | Standard BioTools | 3148022D | AB_2927682 |
|  | 158Gd | E-cadherin | 24E10 | 6.67 | Standard BioTools | 3158029D | AB_2893074 |
|  | 165Ho | β-Catenin | D13A1 | 6.67 | Standard BioTools | 3165032D | AB_2909539 |
|  | 154Sm* | ER | SP1 | 20 | Abcam | ab187260 | AB_2927684 |
|  | 173Yb* | PR | SP2 | 10 | Abcam | ab239793 | AB_2927687 |
|  | 160Gd* | p53 | 7F5 | 20 | Cell signaling | 48818BF | AB_2713958 |
|  | 163Dy | VEGF | G153-694 | 10 | Standard BioTools | 3163028D | AB_2927685 |
|  | 168Er | Ki-67 | B56 | 5 | Standard BioTools | 3168022D | AB_2811061 |
|  | 171Yb | pERK1/2 | D13.14.4E | 9.1 | Standard BioTools | 3171021D | AB_2927686 |
|  | 175Lu | pS6 | N7-548 | 6.67 | Standard BioTools | 3175031D | AB_2864737 |
|  | 172Yb | Cleaved caspase 3 | 5A1E | 20 | Standard BioTools | 3172027D | AB_2928048 |
|  | 143Nd | Vimentin | D21H3 | 2.5 | Standard BioTools | 3143027D | AB_2811069 |
| Cancer stem cell | 147Sm | SOX9 | P-Tyr-100 | 9.1 | Standard BioTools | 3147022D | AB_2890143 |
|  | 149Sm | CD15 | W6D3 | 9.1 | Standard BioTools | 3149026D | AB_2928037 |
|  | 152Sm* | CD133 | RM1029 | 10 | Abcam | ab284397 | AB_2928040 |
|  | 153Eu | CD44 | IM7 | 9.1 | Standard BioTools | 3153029D | AB_2890141 |
|  | 164Dy* | ALDH1 | EP1933Y | 10 | Abcam | ab215996 | AB_2928046 |
|  | 166Er* | SOX2 | EPR3131 | 20 | Abcam | ab215970 | AB_2928047 |
| Stroma | 141Pr | αSMA | 1A4 | 2.5 | Standard BioTools | 3141017D | AB_2890139 |
|  | 169Tm | Collagen type I | Polyclonal | 5 | Standard BioTools | 3169023D | AB_2810857 |
|  | 167Er* | Podoplanin | D2-40 | 10 | Biolegend | 916606 | AB_2565820 |
|  | 151Eu | CD31 | EPR3094 | 5 | Standard BioTools | 3151025D | AB_2890140 |
| Immune | 156Gd | CD4 | EPR6115 | 6.67 | Standard BioTools | 3156033D | AB_2811051 |
|  | 159Tb | CD68 | KP1 | 6.67 | Standard BioTools | 3159035D | AB_2810859 |
|  | 161Dy | CD20 | H1 | 5 | Standard BioTools | 3161029D | AB_2811016 |
|  | 162Dy | CD8a | D8A8Y | 10 | Standard BioTools | 3162035D | AB_2909535 |
|  | 170Er | CD3 | Poly-C-term | 10 | Standard BioTools | 3170019D | AB_2811048 |
| DNA | 191/193Ir | DNA | - | - | Standard BioTools | 201192A | - |
| * In-house conjugated | | | | | | | |
