# Supplementary material for: Multiplex single‐cell profiling of putative cancer stem cell markers ALDH1, SOX9, SOX2, CD44, CD133 and CD15 in endometrial cancer
Source: Mol Oncol. 2025 Jan 31;19(6):1651–67. doi: 10.1002/1878-0261.13815 (PMC12161474; doi:10.1002/1878-0261.13815)
Supplement: Supplementary file 7 — Table S3. Mean percentage of stem‐like phenotypes in non‐endometrioid biopsies and organoids from total epithelial cells per sample. [file MOL2-19-1651-s001.docx]

| Phenotype | Sample | N of sample with phenotype | Mean % of phenotype per sample [range] | P-value* |
| --- | --- | --- | --- | --- |
| ALDH1 | Biopsy | 6/6 | 16.52 [1.53 – 41.88] | 0.589 |
|  | Organoid | 5/6 | 12.54 [0 – 41.95] |  |
| ALDH1/SOX9 | Biopsy | 6/6 | 0.95 [0.05 – 2.64] | 0.818 |
|  | Organoid | 4/6 | 1.84 [0 – 5.26] |  |
| ALDH1/CD44 | Biopsy | 6/6 | 7.82 [0.46 – 24.14] | 0.485 |
|  | Organoid | 5/6 | 4.27 [0 – 12.01] |  |
| ALDH1/CD44/SOX9 | Biopsy | 6/6 | 1.53 [0.006 – 6.08] | 0.818 |
|  | Organoid | 4/6 | 7.19 [0 – 22.00] |  |
| ALDH1/CD44/CD133 | Biopsy | 4/6 | 0.09 [0 – 0.40] | 0.310 |
|  | Organoid | 4/6 | 2.02 [0 – 7.54] |  |
| CD44 | Biopsy | 6/6 | 19.03 [9.32 – 35.35] | 0.818 |
|  | Organoid | 6/6 | 17.26 [2.72 – 36.03] |  |
| CD44/CD15 | Biopsy | 6/6 | 5.71 [0.02 – 32.78] | - |
|  | Organoid | 2/6 | 4.80 [0 – 27.45] |  |
| CD44/SOX9 | Biopsy | 6/6 | 8.35 [0.55 – 33.07] | 0.485 |
|  | Organoid | 6/6 | 5.65 [0.17 – 27.29] |  |
| SOX2 | Biopsy | 6/6 | 1.96 [0.005 – 7.51] | 0.485 |
|  | Organoid | 5/6 | 0.51 [0 – 1.42] |  |
| SOX9 | Biopsy | 6/6 | 7.36 [1.08 – 21.21] | 0.240 |
|  | Organoid | 6/6 | 4.29 [1.00 – 17.26] |  |
| CD15 | Biopsy | 5/6 | 0.86 [0 – 2.91] | 0.485 |
|  | Organoid | 5/6 | 3.31 [0 – 12.00] |  |
| ALDH1/CD44/SOX2 | Biopsy | 0/6 | 0 | - |
|  | Organoid | 5/6 | 1.15 [0 – 6.18] |  |
| ALDH1/CD44/CD15 | Biopsy | 0/6 | 0 | - |
|  | Organoid | 4/6 | 0.21 [0 – 0.76] |  |
| ALDH1/SOX2 | Biopsy | 0/6 | 0 | - |
|  | Organoid | 4/6 | 0.78 [0 – 3.65] |  |
| ALDH1/CD133 | Biopsy | 0/6 | 0 | - |
|  | Organoid | 3/6 | 4.18 [0 – 20.26] |  |
| CD44/CD133 | Biopsy | 4/6 | 0.86 [0 – 5.10] | - |
|  | Organoid | 0/6 | 0 |  |
| Low | Biopsy | 6/6 | 28.96 [15.13 – 47.27] | 0.818 |
|  | Organoid | 6/6 | 30.00 [3.46 – 52.18] |  |

**Supplementary table 3** Mean percentage of stem-like phenotypes in non-endometrioid biopsies and organoids from total epithelial cells per sample

* Mann-Whitney U test
