# Supplementary material for: Multiplex single‐cell profiling of putative cancer stem cell markers ALDH1, SOX9, SOX2, CD44, CD133 and CD15 in endometrial cancer
Source: Mol Oncol. 2025 Jan 31;19(6):1651–67. doi: 10.1002/1878-0261.13815 (PMC12161474; doi:10.1002/1878-0261.13815)
Supplement: Supplementary file 8 — Table S4. CD44 expression associates with clinicopathological variables. [file MOL2-19-1651-s004.docx]

**Supplementary table 4** CD44 expression associates with clinicopathological variables

| Characteristic | CD44 low,  n=127 n(%) | CD44 high,  n=459 n(%) | P-value |
| --- | --- | --- | --- |
| Age (years) |  |  | 0.007 |
| <66 | 50 (17) | 245 (83) |  |
| ≥66 | 77 (27) | 214 (73) |  |
| Histologic type* |  |  | < 0.001 |
| Endometrioid | 86 (18) | 383 (82) |  |
| Non-endometrioid | 41 (35) | 76 (65) |  |
| Clear cell | 6 (29) | 15 (71) |  |
| Serous | 21 (36) | 37 (64) |  |
| Carcinosarcoma | 9 (35) | 17 (65) |  |
| Undifferentiated/other | 5 (42) | 7 (58) |  |
| FIGO stage |  |  | 0.047 |
| I | 85 (19) | 358 (81) |  |
| II | 13 (28) | 34 (72) |  |
| III | 19 (27) | 50 (73) |  |
| IV | 10 (37) | 17 (63) |  |
| Histologic Grade** |  |  | 0.007 |
| I | 37 (18) | 166 (82) |  |
| II | 21 (13) | 147 (87) |  |
| III | 26 (30) | 61 (70) |  |
| Recurrence |  |  | 0.040 |
| No | 85 (20) | 349 (80) |  |
| Yes | 42 (28) | 110 (72) |  |
| Molecular class |  |  | < 0.001 |
| POLE | 9 (26) | 25 (74) |  |
| MMR-D | 25 (15) | 144 (85) |  |
| CNL | 25 (17) | 125 (83) |  |
| CNH | 36 (38) | 58 (62) |  |

* Comparing endometrioid vs. non-endometrioid

** Endometrioid only

Missing information regarding histologic grade for 8 patients, molecular class for 139 patients
